# Quackery

**Published:** 1844-11

**Authors:** 


*Quackery.*—A fatal case of empirical treatment lately took place in Wurtsboro', (N. Y. we suppose), an infusion of tobacco and the entrails of some animal having been, it is said the active agents in the hands of the quack whose name was John Hollister.—*Ibid.*

